# BATF3 is sufficient for the induction of *Il9* expression and can compensate for BATF during Th9 cell differentiation

**DOI:** 10.1038/s12276-019-0348-6

**Published:** 2019-11-27

**Authors:** Woo Ho Lee, Sung Woong Jang, Hyeong Su Kim, So Hee Kim, Jung In Heo, Ga Eul Kim, Gap Ryol Lee

**Affiliations:** Department of Life Science, Sogang University, 35 Baekbeom-ro, Mapo-gu Seoul, 04107 Korea

**Keywords:** CD4-positive T cells, Lymphocyte differentiation

## Abstract

Th9 cells preferentially produce IL-9 and participate in allergic responses and asthma. Differentiation of Th9 cells is induced by IL-4 and TGF-β, and then the cells are amplified by OX40 signals. The transcription factors PU.1, IRF4, and BATF are required for Th9 differentiation. BATF3 is an AP-1 family transcription factor that is highly homologous to BATF; however, its role in Th9 cells is poorly defined. Here, we show that OX40 signaling induced the expression of *Batf3* and that its overexpression in the presence or absence of OX40 signaling increased the expression of IL-9 in Th9 cells. BATF3 physically interacted with IRF4 and was bound to the *Il9* locus. A transient reporter assay revealed that the BATF3–IRF4 complex induced *Il9* promoter activity. BATF3 rescued *Il9* expression and restored the capacity to induce the airway inflammation in *Batf* KO Th9 cells. Thus, BATF3 itself is sufficient for the induction of Th9 cell differentiation and can substitute for BATF during Th9 cell differentiation.

## Immunology: fanning the fires of airway inflammation

A protein that regulates gene expression in immune cells may contribute to airway inflammation in asthma and allergies. A subset of immune cells known as Th9 cells plays a prominent role in these respiratory disorders, and researchers led by Gap Ryol Lee at Sogang University in Seoul, South Korea, set out to characterize signaling mechanisms that promote Th9 production. They focused specifically on a protein called BATF3, which regulates genes responsible for maturation of a variety of other immune cell types, but whose role with regard to Th9 is poorly understood. They determined that BATF3 has a potent effect in terms of inducing Th9 cell activity, and can even take the place of another known Th9-inducing protein. These findings could assist in the development of treatments that keep airway-constricting inflammation in check.

## Introduction

CD4^+^ T cells are essential for coordinating immune responses. These cells are comprised of several subsets, which are categorized as conventional T (Tconv) cells and regulatory T (Treg) cells. Tconv cells, comprising T helper type 1 (Th1), Th2, Th9, Th17, and T follicular helper cells, activate immune responses^1^. By contrast, Treg cells suppress immune responses. The balance between Tconv cells and Treg cells is important for maintaining immune homeostasis^2,3^. Recent studies show that Th9 cells are a subset of CD4^+^ T cells that mainly secrete interleukin (IL)-9^4,5^. Differentiation of Th9 cells from naive CD4 T cells requires cytokines IL-4 and transforming growth factor beta (TGF-β), along with transcription factors STAT6, PU.1, IRF4, ETV5, and GATA3^4–7^. Th9 cells play an important role in allergic inflammation, autoimmune disease, and tumor immunity^6,8–10^.

Basic leucine zipper transcriptional factor ATF-like (BATF) and basic leucine zipper transcription factor ATF-like 3 (BATF3) both lack a transcription activation domain, so they activate target genes by interacting with interferon regulatory factor (IRF) family proteins. The IRF family comprises nine members (IRF1 to IRF9), all of which have a DNA-binding domain^11^. The c-Rel/NF-κB complex in CD4^+^ T cells, which is induced by T cell receptor stimulation, binds to the *Irf4* promoter and induces expression of *Irf4*^12^. IRF4 regulates all subsets of CD4^+^ T cells. In Th2 cells, IRF4 binds to the *Il4* gene promoter, thereby increasing its expression^13^. In addition, IRF4 upregulates GATA3, a key transcription factor in Th2 cells^14^. In Th9 cells, IRF4 acts similarly as in Th2 cells, i.e., it induces Th9 cell differentiation by binding to the *Il9* promoter, and its deficiency inhibits Th9 cell differentiation^6^.

BATF3 is an activator protein (AP)-1 family transcription factor that is highly homologous with BATF^15^. Both BATF and BATF3 comprise only two domains: a DNA-binding domain and a leucine zipper motif. These transcription factors compensate for each other’s function in dendritic cells and Th17 cells^15^. BATF is required for Th9 cell differentiation because it induces the IL-9 production through cooperative binding with IRF4 to the *Il9* promoter^16^. *Batf*-deficient CD4^+^ T cells show a reduced capacity to induce allergic inflammation, whereas the opposite is true for *Batf*-overexpressing Th9 cells. BATF3 is important for dendritic cell development and for Treg cell differentiation^17,18^.

OX40 ligand (OX40L), also called tumor necrosis factor superfamily member 4, is a ligand for OX40 (CD134) that is expressed only by antigen-presenting cells (APCs). In addition, OX40 is expressed only by activated CD4^+^ and CD8^+^ T cells^19–21^. OX40L signals provided by APCs serve as a costimulatory signal that amplifies and sustains activated T cells by increasing the production of cytokines that stimulate T cell proliferation^22^. A recent study showed that OX40L–OX40 signaling activates TRAF6 and then induces the NF-κB signaling pathway^23^. OX40L–OX40 signaling promotes the expression of BATF3 in Treg cells^24,25^.

Here, we investigated the role of BATF3 in Th9 cells stimulated by OX40L. We found that the expression of *Batf3* in Th9 cells increased in the presence or absence of OX40L signaling. When *Batf3* was overexpressed in Th9 cells, IL-9 production increased. In addition, BATF3 interacted physically with IRF4; the resulting BATF3–IRF4 complex increased *Il9* promoter activity. BATF3 could compensate for BATF during Th9 differentiation and induction of airway inflammation in *Batf* knockout (KO) mice. These results suggest that BATF3 plays an important role in Th9 differentiation and allergic inflammation.

## Materials and methods

### Mice

All mice were on a C57BL/6 background. Wild-type (WT) mice (aged 6–8 weeks) were purchased from Daehan Bio Link. *Batf* KO, *Batf3* KO, and *Rag1* KO mice were purchased from the Jackson laboratory. All mice were raised under specific pathogen-free conditions, and all animal experiments were approved by the Sogang University Institutional Animal Care and Use committee.

### ICS

Cells were stimulated for 4 h with PMA (50 ng/ml) and ionomycin (1 μM; both from Sigma-Aldrich) in the presence of Brefeldin A (BioLegend). Cells were fixed and permeabilized using Cytofix/Cytoperm and Perm/Wash buffer (BD Biosciences). A PE-conjugated anti-IL-9 antibody (BioLegend) was used for IL-9 cytokine staining. Stained cells were analyzed using an Accuri C6 plus cytometer (BD Biosciences).

### Purification of CD4^+^ T cells and stimulation in vitro

Naive CD4^+^ T cells were isolated from mouse spleens, using a MojoSort Ms CD4^+^ naive T cell Isolation Kit (BioLegend, Cat. No. 480040) according to the manufacturer’s recommendations. In brief, red blood cells were removed from splenocytes, using ACK lysis buffer. A mouse naive CD4^+^ T cell biotin–antibody cocktail was used for negative selection. In brief, after incubation on ice for 15 min, 1.0 × 10^7^ cells were treated with 10 µl of streptavidin nanobeads. Naive CD4^+^ T cells were cultured in RPMI-1640 medium supplemented with 5% fetal bovine serum, 2-mercaptoethanol, MEM amino acids, nonessential MEM amino acids, and penicillin/streptomycin (all supplemented from Gibco Life Technologies), and they were activated by plate-bound anti-CD3 (10 µg/ml) and anti-CD28 (2 µg/ml) antibodies. To induce Th0 cells, the cells were exposed to mouse recombinant IL-2 (1 ng/ml, eBioscience) for 3 days. To induce differentiation into Th9 cells, cultures were treated for 3 days with anti-IFN-γ (5 µg/ml, R&D), mouse recombinant IL-2 (0.2 ng/ml), human recombinant TGF-β1 (2 ng/ml, R&D), mouse recombinant IL-4 (40 ng/ml, R&D), and an anti-IL10 receptor antibody (1 µg/ml, eBioscience). Finally, Th9 cells were exposed to OX40L (100 ng/ml) and an anti-His tag antibody (20 µg/ml) under Th9 + OX40L conditions.

### Retroviral transduction

To generate overexpression constructs, mouse complementary DNA encoding *Batf* or *Batf3* was cloned into a MIEG3 retroviral vector. A total of 1.8 × 10^6^ Phoenix Eco cells were transfected with the empty MIEG3 vector, MIEG3-*Batf*, or MIEG3-*Batf3*, along with a pCL-Eco helper vector. After transfection, cells were cultured for 2 days, and supernatants were collected. Naive CD4^+^ T cells were isolated from mouse spleens and were cultured for 24 h under Th9 or Th9 + OX40L conditions. Activated T cells were spin-infected (1600 *g* for 90 min at 30 °C) with 1 ml of supernatant containing retrovirus and polybrene (4 µg/ml). After infection, cells were cultured for 2 days under Th9 or Th9 + OX40L conditions. After another 2 days, cells were either stimulated with PMA and ionomycin for 4 h before intracellular cytokine analysis, or green fluorescent protein (GFP)^+^ cells were sorted and used for RNA quantification.

### RNA isolation and qRT-PCR

RNA was isolated from cells using TRIZol reagent (Molecular Research Center, Inc.). Quantitative reverse transcription polymerase chain reaction (RT-PCR) was performed with TOPscript RT (Enzynomics), qPCRBIO Blue Mix Lo-ROX (PCR Biosystems), and qPCRBIO Probe Mix Lo-ROX (PCR Biosystems), using a LightCycler 96 instrument (Roche).

### ChIP assay

Naive CD4^+^ T cells were cultured for 3 days under Th9 conditions and were then harvested. A Magna ChIP A/G kit (Sigma-Aldrich) was used for chromatin immunoprecipitation (ChIP) assays. In brief, cells were fixed for 10 min at room temperature in 1% formaldehyde (1 ml per 1.0 × 10^6^ cells). Next, 10× glycine buffer was added to the cell suspension at room temperature for 10 min to quench the fixation reaction. Harvested cells were resuspended in cell lysis buffer containing Protease Inhibitor Cocktail II (Sigma-Aldrich). Lysed cells were sonicated at 60 W (30 s on, 30 s off) for 4 min. Immunoprecipitation was performed by adding 1 µg of control IgG (Santa Cruz Biotechnology) or 2 µg of anti-FLAG antibody (Sigma-Aldrich) to the lysate. All qRT-PCR reactions were performed in triplicate using qPCRBIO SyGreen Blue Mix Lo-ROX. The primer sequences are listed in Table S1^26,27^.

### Immunoblot analysis

Proteins in cell lysates were separated by sodium dodecyl sulfate (SDS)/polyacrylamide gel electrophoresis and transferred to a PVDF membrane. The membrane was blocked in 5% skim milk/TBST buffer, which was followed by incubation overnight at 4 °C with an anti-IRF4 antibody (diluted 1:1,000 in 5% skim milk). Next, the membrane was washed with TBST buffer and incubated for 1 h at room temperature with an HRP-conjugated anti-goat IgG antibody (diluted 1:1,000 in 5% skim milk). Finally, the membrane was washed with TBST buffer and incubated with an enhanced chemiluminescence substrate.

### ELISA

A mouse IL-9 uncoated ELISA Kit (Invitrogen) was used according to the manufacturer’s recommendations. In brief, a coating buffer containing a capture antibody (100 µl/well) was added to coat a Corning Costar 9018 enzyme-linked immunosorbent assay (ELISA) plate. The sealed plate was incubated overnight at 4 °C. The coating buffer was removed, and the plate was washed with wash buffer. Each well was filled with ELISA–enzyme-linked immune absorbent spot (ELISPOT) diluent (1 × 200 µl/well) for 1 h at room temperature to block nonspecific binding. After incubation, the wells were washed and loaded with ELISA/ELISPOT diluent (50 µl/well). Next, standards (50 µl/well) and samples were added to the wells, and the plates were sealed and incubated overnight at 4 °C. Standards and samples were removed, and the plate was washed three times. Next, a detection antibody (100 µl/well) was added to all wells and incubated for 1 h at room temperature. After washing three times, avidin-HRP (100 µl/well) was added to the plate for 30 min at room temperature. To initiate the color reaction, 1× TMB solution (100 µl/well) was added to each well. After 15 min, the reaction was stopped by the addition of 1 M H_2_SO_4_ (50 µl/well). The plate was read at 450 nm in an iMark microplate reader (Bio-Rad).

### Co-IP

HEK293T cells were transfected with pCMV-*Batf3-Flag* and pCMV-*Irf4* constructs. After 2 days, cells were harvested, washed in PBS, and dissolved in IP 150 buffer. Cells were sonicated to obtain a cell lysate and were then precleared by incubation with protein A/G (Santa Cruz). Precleared lysates were treated with an anti-FLAG antibody plus agarose or with normal IgG, and then incubated overnight at 4 °C. Normal IgG-treated samples were precipitated with protein A/G. After 2 h, the samples were washed and incubated with SDS loading dye before immunoblot analysis.

### Transient reporter assay

The *Il9* promoter was cloned into a pGL3 vector (Promega). EL4 cells were transfected with the reporter construct, a pRL vector, and transcription factor expression vectors by electroporation (260 V, 950 µF). Next, cells were cultured for 1 day in DMEM (Welgene) containing 10% FBS and 1% antibiotics. Next, cells were stimulated for 4 h with PMA (50 ng/ml) and ionomycin (1 µM) before lysis in passive lysis buffer. Luciferase activity was measured in an LB96V luminometer (Berthold).

### Adoptive transfer model for assessment of airway inflammation

On days 0 and 7, *Batf* KO mice were sensitized with ovalbumin (OVA; 20 µg) and 100 µl of aluminum hydroxide gel via intraperitoneal injection. On day 14, CD4^+^ T cells were isolated from the mice and cultured (1 × 10^6^ cells/plate) for 24 h under Th0 conditions. Next, the cells were transduced with an empty vector or a Batf3 expression vector as described above and were cocultured with WT splenocytes (CD4^+^ T cells were removed; 1 × 10^7^ cells/plate) for 3 days in the presence of OVA (200 µg/ml) under Th9-inducing conditions. Cells were washed twice with PBS and were then suspended in 100 µl of PBS. Cells were then injected intraperitoneally into *Rag1* KO mice. After 3 days, recipient mice were challenged (for 40 min each time) for 4 days with an aerosol that was 1% OVA in PBS. Mice were sacrificed 24 h after the final challenge.

### Analysis of BAL fluid

Bronchoalveolar lavage (BAL) fluid isolated from recipient mice was centrifuged for 5 min at 1000 *g*. Supernatants were then collected and stored at −80 °C before ELISAs. Cells were then resuspended in 100 µl of PBS (10 µl of cell suspension diluted in 100 µl of PBS) and were counted. The remaining cells were collected with a cytospin and stained with Diff Quik (Sysmex). The morphology and staining characteristics of at least 150 cells were assessed.

### RNA isolation and histology of lung tissue

Lungs were isolated from recipient mice for RNA isolation and histological analysis. One part of the lung was homogenized in TRIZol reagent before RNA isolation. The other part was fixed in 4% formaldehyde solution and incubated at 4 °C to remove any remaining blood. After 24 h, the lung tissue was transferred to a fresh 4% formaldehyde solution. Lung sections were stained with periodic acid Schiff (PAS) reagent to assess mucus production, or they were stained with hematoxylin and eosin (H&E) to detect infiltrating cells.

### Analysis of serum IgE

Blood obtained from mouse hearts was incubated on a rotator overnight at room temperature. Blood was centrifuged at 16,000 *g* for 15 min, and serum was stored at −80 °C for an IgE ELISA.

## Results

### OX40 signaling increases the expression of *Batf3* in Th9 cells

**Fig. 1:**
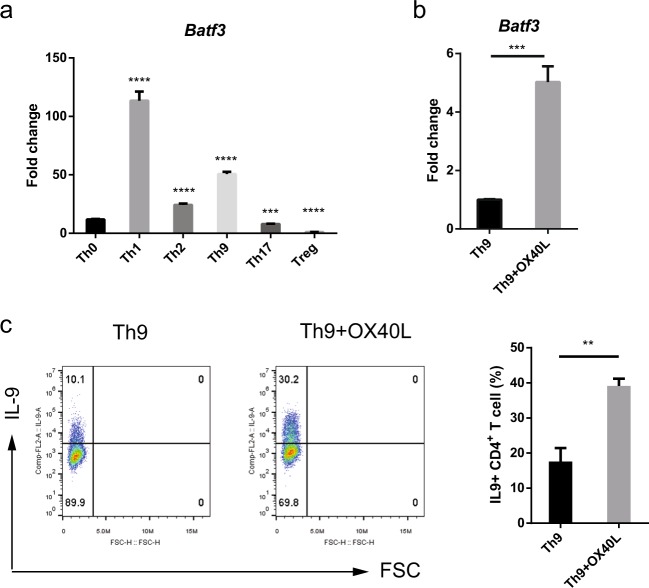
*Batf3* expression in Th9 cells increases significantly upon exposure to OX40L. **a** Expression of *Batf3* messenger RNA (mRNA) in cells stimulated under Th0, Th1, Th2, Th9, Th17, and Treg conditions was measured by qRT-PCR. Data were normalized to *Gapdh* expression and are shown relative to Th0 conditions. Statistical analyses were performed by comparing Th0 with each subset. **b** Expression of *Batf3* mRNA in cells stimulated under Th9 or Th9 + OX40L conditions (with OX40L under Th9 conditions) was measured by qRT-PCR. Data were normalized to those of *Gapdh* and relative to Th9 conditions. **c** Naive CD4^+^ T cells were cultured for 3 days under Th9 or Th9 + OX40L conditions, and the percentage of IL-9^+^ CD4^+^ T cells was measured by flow cytometry. Left: data are representative of three independent experiments. Right: data are expressed as the mean of three independent experiments. The error bars represent the s.d. *P* values were calculated using Student’s *t*-tests. *P < 0.05, **P < 0.01, ***P < 0.001, and ****P < 0.0001.

To study the role of BATF3 in Th9 cells, we first examined its expression. Naive CD4 T cells were differentiated into various subsets, and the expression of *Batf3* was measured by qRT-PCR. The expression of *Batf3* in Th9 cells was relatively high compared to other subsets, with the exception of Th1 cells (Fig. 1a). When naive CD4^+^ T cells were differentiated into Th9 cells in the presence of OX40L (herein described as Th9 + OX40L conditions), the expression of *Batf3* and IL-9 increased markedly (Fig. 1b, c); this result is consistent with observations from previous studies^24,25^. These data suggest that OX40L increases the expression of *Batf3* and IL-9 in Th9 cells.

### Overexpression of *Batf3* increases the production of IL-9 in Th9 cells

**Fig. 2:**
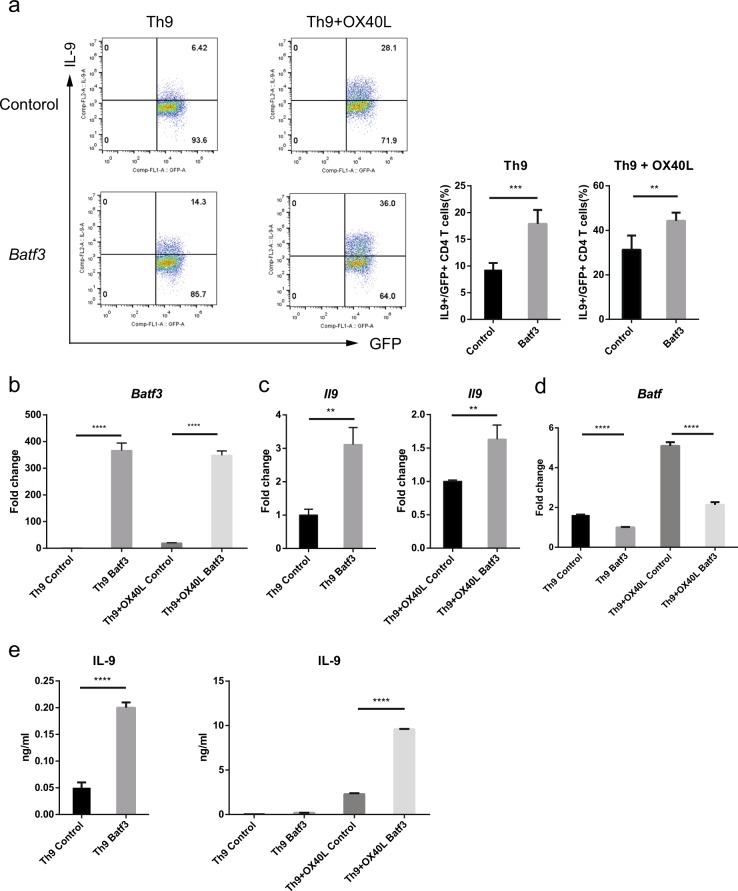
Increased expression of IL-9 in *Batf3-*overexpressing Th9 cells. **a** CD4^+^ T cells were cultured under Th9 or Th9 + OX40L conditions. Transduced cells were gated for GFP and analyzed by flow cytometry. **b**, **c** Transduced cells were sorted using a flow cytometer, and RNA was isolated. The expression of *Batf3* and *Il9* mRNA under each condition was measured by qRT-PCR. Data were normalized to *Gapdh* levels and are shown relative to Th9 control or Th9 + OX40L control cells. **d** Expression of *Batf* mRNA was measured by qRT-PCR when *Batf3* was overexpressed under each condition. **e** Cells were stimulated for 4 h with PMA and ionomycin. The concentration of IL-9 in the culture supernatant was measured by ELISA. Error bars represent the s.d. *P* value were calculated using Student’s *t*-tests. *P < 0.05, **P < 0.01, and ***P < 0.001. All experiments were repeated independently three or four times.

To examine the role of BATF3 in Th9 cells, we overexpressed *Batf3* by transducing cells with a MIEG3-*Batf3* retroviral construct and then performed flow cytometric analysis. The MIEG3 retroviral vector contains a gene that encodes GFP; therefore, transduced cells were identified and sorted by flow cytometry. When *Batf3* was overexpressed in Th9 cells, IL-9 production increased both in the absence and presence of OX40L (Fig. 2a). The expression of *Batf3* and *Il9* in transduced cells was confirmed by qRT-PCR (Fig. 2b, c). The level of *Il10* expression was slightly increased by *Batf3* overexpression, but it was very low compared to *Il9* expression levels (Fig. S1). The expression of *Batf* was decreased by *Batf3* overexpression in Th9 cells regardless of OX40L stimulation (Fig. 2d). Next, we performed ELISAs to measure IL-9 secretion from control and *Batf3*-overexpressing Th9 cells. Transduced cells were stimulated under Th9 polarizing conditions and were then restimulated for 4 h with PMA and ionomycin. The amount of IL-9 protein in the supernatant increased when *Batf3* was overexpressed; it increased further upon exposure to OX40L (Fig. 2e). These data show that overexpression of *Batf3* affects the expression of IL-9 in Th9 cells and that OX40L amplifies IL-9 expression. We also investigated whether *Batf3* has a role in the other subsets of CD4 T cells, using the overexpression model (Fig. S2). *Batf3* overexpression inhibited Th1, Th2, and Treg differentiation but had no effect on Th17 differentiation (Fig. S2). Thus, it seems that Batf3 enhances only Th9 differentiation (Fig. S2).

### The BATF3–IRF4 complex increases *Il9* promoter activity

**Fig. 3:**
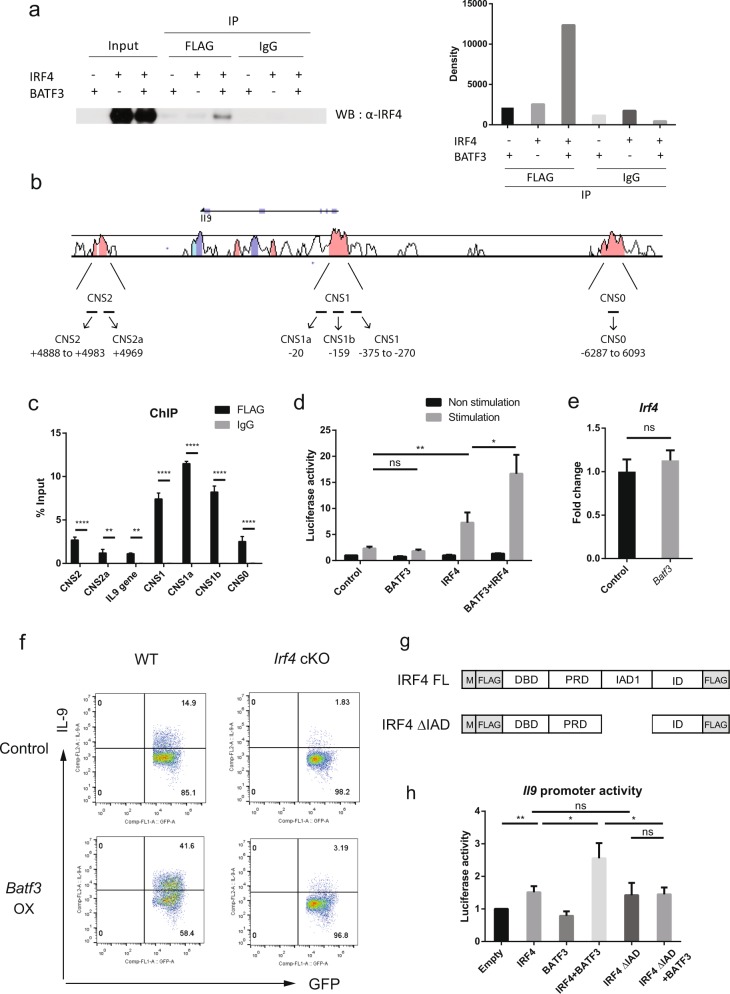
BATF interacts physically with IRF4 and increases *Il9* promoter activity by binding to the CNS1 within the *Il9* locus. **a** BATF3-FLAG and IRF4 were expressed in HEK293T cells. Cell lysates were precipitated by an anti-FLAG antibody plus agarose or by a control IgG antibody. Precipitates were analyzed by immunoblotting (left), and the immunoblot was quantified by densitometry (right). **b** The CNSs of the *Il9* locus. The numbers indicate the binding sites of the primers. **c** BATF3-binding sites within the CNSs of *Il9* were analyzed in a ChIP assay. Control IgG and anti-FLAG antibodies were used for immunoprecipitation, and qPCR was performed for quantification. **d** *Il9* promoter activity was measured in a transient reporter assay. EL4 cells were transfected with pCMV expression vectors. Cells were stimulated for 4 h with PMA and ionomycin, and were then analyzed in a luminometer. **e** Expression of *Irf4* mRNA was measured by qRT-PCR under control or *Batf3*-overexpressing conditions. Data were normalized to the levels of *Gapdh* and are shown relative to controls. **f** Naive CD4^+^ T cells from WT and *Irf4* cKO mice were transduced with an empty or *Batf3* overexpression vector. The cells were stimulated under Th9 conditions for 3 days. Transduced cells were gated for GFP, and then IL-9 production was measured by flow cytometry. OX, overexpression. **g** Diagram of the IAD domain deletion mutant of IRF4. **h** *Il9* promoter activity was measured by a transient reporter assay. EL4 cells were transfected with CMV vectors expressing the indicated transcription factors and were then stimulated with PMA and ionomycin for 4 h. Error bars represent the s.d. *P* values were calculated using Student’s *t*-tests. *P < 0.05, **P < 0.01, and ***P < 0.001. All experiments were repeated independently three times.

To identify the molecular mechanism underlying the observed *Batf3*-mediated increase of *Il9* expression in Th9 cells, we tried to identify proteins that interact with BATF3. Previous reports show that IRF4 binds to BATF^28^, and that BATF and BATF3 are highly homologous and can compensate for each other in immune cells^15^; therefore, we considered the possibility that BATF3 may bind to IRF4. We tested this in coimmunoprecipitation (co-IP) experiments involving BATF3- and IRF4-expressing HEK293T cells. The results revealed that BATF3 and IRF4 interact with each other (Fig. 3a). Next, we performed a ChIP assay to determine whether BATF3 binds to the conserved noncoding sequences (CNSs) of the *Il9* locus (Fig. 3b). BATF3 bound to several CNSs within the *Il9* locus. In particular, BATF3 bound to CNS1 (Fig. 3c). The binding of IRF4 to CNSs within the *Il9* locus was reported previously^26,27^. CNS1 includes the *Il9* gene promoter, and it was bound by IRF4^6,27^. BATF family members cooperate with IRF4 by physically interacting with IRF4, binding to the AP-1-IRF composite elements (AICEs) in their target genes and regulating the genes^28–30^. Therefore, we used a transient reporter assay to examine whether the BATF3–IRF4 complex affects *Il9* promoter activity. Thus, we cloned the *Il9* promoter into the promoterless pGL3 vector; the promoter sequence was from −1156 to +17 bp and contained the CNS1 locus (−610 to +17) and AICE motif (−238 bp). We found that BATF3 alone did not alter *Il9* promoter activity; however, IRF4 alone somewhat increased *Il9* promoter activity (Fig. 3d). When BATF3 and IRF4 were expressed together, they synergistically enhanced *Il9* promoter activity (Fig. 3d). This increase was not simply due to the increased amount of IRF4, since the expression of *Irf4* was not different between control and *Batf3-*overexpressing Th9 cells (Fig. 3e). To determine whether the formation of the BATF3 and IRF4 complex is required for transcriptional activation, *Irf4* fl/fl CD4-Cre (*Irf4* cKO) mice were used. *Batf3* overexpression failed to induce IL-9 in *Irf4*-deficient Th9 cells (Fig. 3f), suggesting that IRF4 is essential for IL-9 expression. We also made a truncated form of IRF4, which lacks the IRF association domain (IAD; 238–410 bp among the 450 bp full-length IRF4; Fig. 3g). IAD is known to be a protein–protein interaction domain^28,31,32^. IRF4 ΔIAD alone could induce Il9 promoter activity to the same level that full-length IRF4 could (Fig. 3h), probably because it contains a DNA-binding domain (N-terminal) and a regulatory domain (C-terminal). However, when coexpressed with BATF3, IRF4 ΔIAD did not have any synergistic effect (Fig. 3h), suggesting that the synergistic effect of BATF3 and IRF4 requires their interaction via the IRF4 IAD. Taken together, these data show that BATF3 interacts with IRF4 and that the BATF3–IRF4 complex acts synergistically to increase *Il9* promoter activity to facilitate Th9 differentiation.

### BATF and BATF3 compensate for each other in Th9 cells

**Fig. 4:**
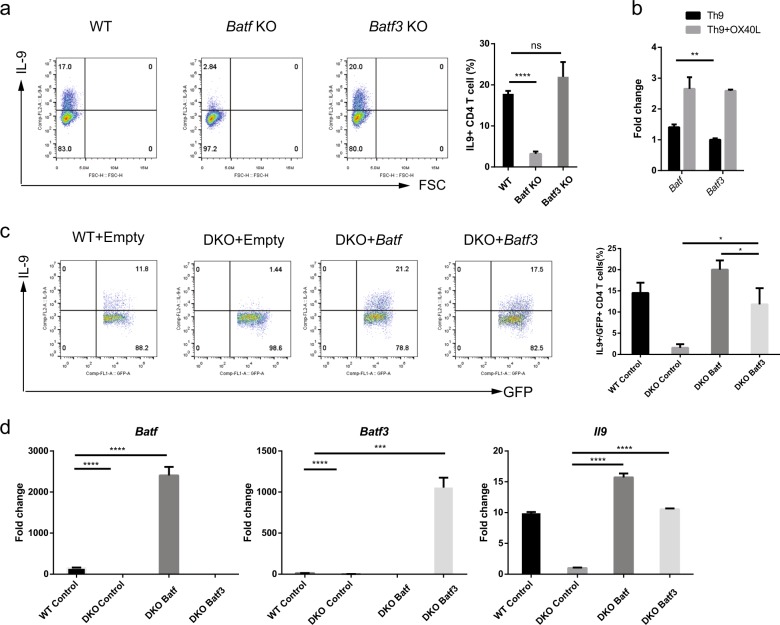
BATF and BATF3 play redundant roles and compensate for each other. **a** Naive CD4^+^ T cells from WT, *Batf* KO, and *Batf3* KO mice were cultured for 3 days under Th9 + OX40L conditions, and were then stimulated for 4 h with PMA and ionomycin. IL-9 production by cells was measured by flow cytometry. **b** Expression of *Batf* and *Batf3* mRNA in the presence or absence of OX40L. **c** Naive CD4^+^ T cells were isolated from the spleens of WT or *Batf-Batf3* DKO mice and then cultured for 3 days under Th9 + OX40L conditions. Cells were transfected with *Batf* or *Batf*3 expression constructs. Transduced cells were gated for GFP, and IL-9 production was measured by flow cytometry. **d** Transduced cells were sorted by flow cytometry, and RNA was isolated. The expression of *Batf*, *Batf3*, and *Il9* mRNA was measured by qRT-PCR. Error bars represent the s.d. *P* values were calculated using Student’s *t*-tests. *P < 0.05, **P < 0.01, and ***P < 0.001. All experiments were repeated independently three times.

Next, we asked whether IL-9 production by CD4^+^ T cells decreased in the absence of *Batf3*. Naive CD4^+^ T cells from WT, *Batf* KO, and *Batf3* KO mice were stimulated under Th9-polarizing conditions in the presence of OX40; the percentage of IL-9-expressing cells was then measured by flow cytometry. The percentage of IL-9-expressing cells in *Batf3* KO mice was not decreased; however, the number in *Batf* KO mice decreased markedly (Fig. 4a). We also investigated whether *Batf3* had a role in the other subsets of CD4 T cells when *Batf3* was deleted (Fig. S3). *Batf3* deletion caused enhanced Th2 and Treg differentiation, but did not influence Th1 and Th17 differentiation (Fig. S3).

The result that *Batf3* KO cells did not reduce IL-9 expression was unexpected and somewhat puzzling because overexpression of *Batf3* induced IL-9 production in Th9 cells (Fig. 2). Therefore, we hypothesized that BATF and BATF3 play a redundant role, and that BATF compensates for the loss of BATF3. BATF is an important transcription factor in Th9 cells^16^, and BATF and BATF3 compensate for each other in dendritic cells^15,28^. To test this hypothesis in Th9 cells, we first measured *Batf* expression under Th9-polarizing conditions in the presence or absence of OX40 (Fig. 4b). *Batf* was expressed in Th9 cells, and its expression was increased in the presence of OX40L as *Batf3*. This result suggests that *Batf* expression was sufficient to compensate for *Batf3*. Second, we generated *Baft–Batf3* double KO (DKO) mice. DKO naive CD4^+^ T cells were transduced with a *Batf* or a *Batf3* expression vector and cultured under Th9 + OX40L conditions. As expected, the percentage of DKO cells expressing IL-9 was much lower than the number of WT cells expressing IL-9 (Fig. 4c). Reconstitution of BATF or BATF3 restored the expression of IL-9 in DKO Th9 cells, although the BATF3 restored levels were not as high (Fig. 4c). We confirmed the expression of *Il9*, *Batf*, and *Batf3* at the RNA level (Fig. 4d). These data support the hypothesis that BATF and BATF3 play a redundant role in Th9 cells, and that they compensate for each other when necessary.

### BATF3 induces the expression of *Il9* in *Batf* KO Th9 cells

**Fig. 5:**
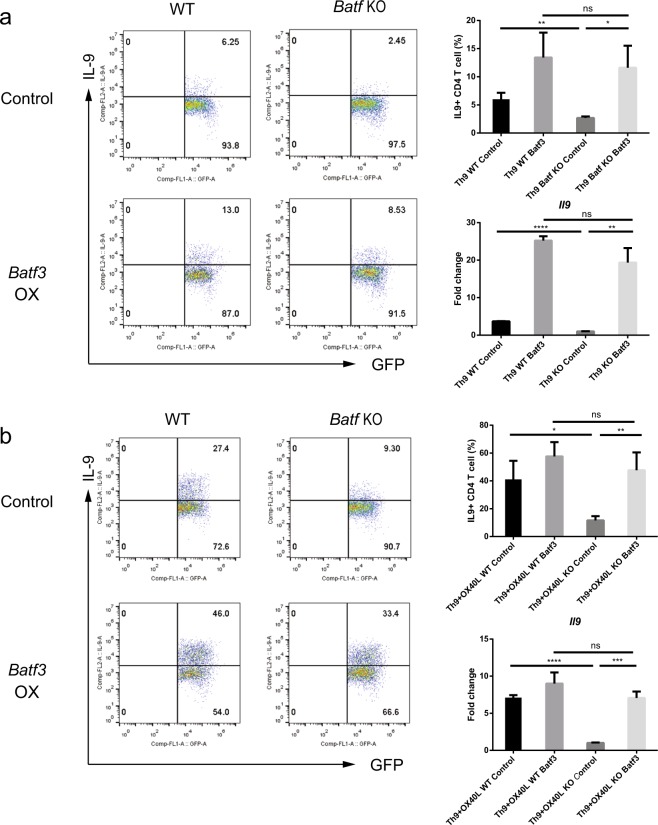
BATF3 can compensate for the loss of BATF and induce *Il9* expression. **a**, **b** *Batf* KO naive CD4^+^ T cells were transduced with a retroviral construct and cultured under Th9 (**a**) or Th9 + OX40L (**b**) conditions. Transduced cells were gated for GFP, and IL-9 production was measured by flow cytometry. In addition, transduced cells were sorted by flow cytometry before RNA isolation. Expression of *Il9* mRNA in cells stimulated under Th9 (a) or Th9 + OX40L (**b**) conditions was measured by qRT-PCR. Data were normalized to levels of *Gapdh* and are shown relative to KO controls under both conditions. OX, overexpression. Error bars represent the s.d. *P* values were calculated using Student’s *t*-tests. *P < 0.05, **P < 0.01, and ***P < 0.001. All experiments were repeated independently three times.

To delineate the role of BATF3 in Th9 cells, we used *Batf* KO mice, which are defective in *Il9* expression. We asked whether overexpression of *Batf3* induces the production of IL-9 in *Batf* KO Th9 cells. BATF3 restored IL-9 levels in *Batf* KO Th9 cells to levels that were similar to those observed in WT cells, either in the absence or presence of OX40L (Fig. 5a, b). Restoration in *Batf* KO cells was confirmed at the RNA level, and it was consistent with the intracellular cytokine staining (ICS) data (Fig. 5a, b). Therefore, BATF3 increases and restores the production of IL-9, thereby compensating for the loss of BATF in Th9 cells.

### *Batf3*-overexpressing Th9 cells induce airway inflammation, even in the absence of BATF

**Fig. 6:**
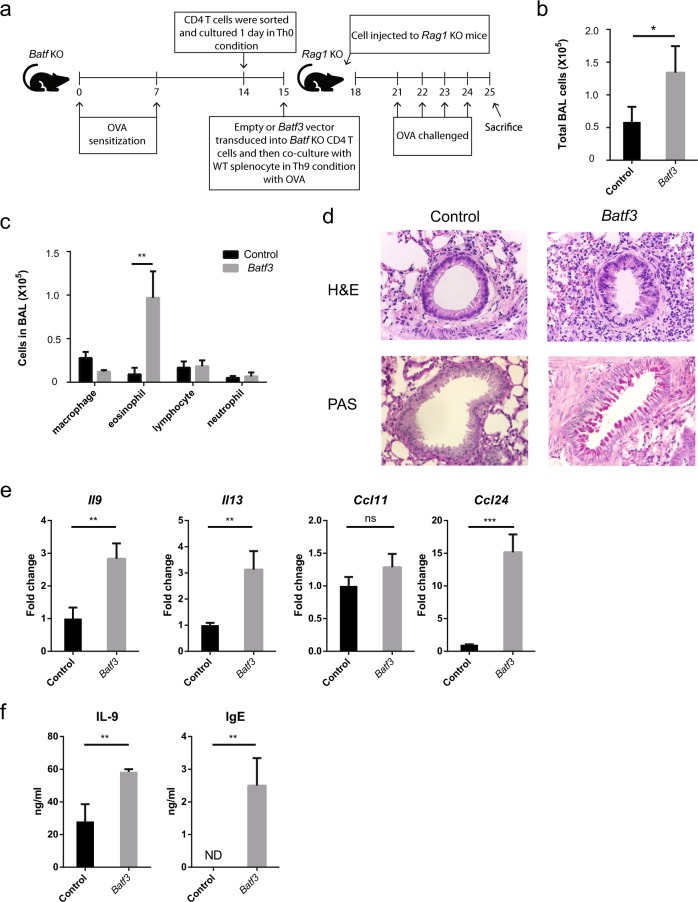
*Batf* KO Th9 cells induce airway inflammation upon overexpression of *Batf3*. **a** Schematic diagram of the adoptive transfer model used to induce airway inflammation. Naive CD4^+^ T cells from OVA-sensitized *Batf* KO mice were isolated and cocultured with WT APCs under Th9 conditions in the presence of OVA. Next, the cells were adoptively transferred (intraperitoneally) into *Rag1* KO mice. Recipient mice were challenged with an OVA aerosol. **b** The total number of BAL cells in the lungs of the mice was counted using a hemocytometer. **c** Cells were stained with Diff Quik to identify different cell types. Next, at least 150 cells were counted per sample. **d** Lung tissues were stained with H&E and PAS before histological analysis. The top panels show cells infiltrating the lung. The bottom panels show mucus production. **e** Expression of mRNA encoding asthma-associated genes was measured by qRT-PCR. Data were normalized to levels of *Gapdh* and are shown relative to control cells. **f** Secretion of IL-9 protein into BAL fluid and IgE levels in mouse serum were measured by ELISA. Error bars represent the s.d. *P* values were calculated using Student’s *t*-tests. *P < 0.05, **P < 0.01, and ***P < 0.001. All experiments were repeated independently three times.

*Batf-*deficient CD4^+^ T cells cannot induce airway inflammation in *Rag1* KO mice^16^. Here, we investigated whether BATF3 can substitute for BATF in the induction of airway inflammation. For this, we used an adoptive transfer model (Fig. 6a)^33^ in which OVA-sensitized *Rag1* KO mice received OVA-stimulated *Baft3*-overexpressing *Batf* KO Th9 cells, followed by challenge with OVA. BAL cells were collected from the lungs of mice and analyzed by cell counting and differential cell staining. The total cell number in BAL fluid from mice that received *Batf3*-overexpressing *Batf* KO Th9 cells increased (Fig. 6b). In addition, *Batf3* overexpression increased the recruitment of eosinophils (Fig. 6c). Cell infiltration and mucus production in the lung, as measured by H&E and PAS staining, respectively, increased upon overexpression of *Batf3* (Fig. 6d). Consistent with these data, we found that expression was increased for *Il9*, *Il13*, and *Ccl24*, but not *Ccl11*, in the lung upon overexpression of *Batf3* (Fig. 6e). Moreover, the levels of IL-9 protein in BAL fluid and IgE in serum increased upon *Batf3* overexpression (Fig. 6f). Taken together, these data suggest that *Batf* KO Th9 cells can induce airway inflammation upon overexpression of *Batf3* in an animal model of asthma and that BATF3 compensates for the loss of BATF.

## Discussion

BATF and BATF3 are important transcription factors in immune cells. Previous studies examined the role of BATF3 in dendritic cells and the role of BATF in CD4^+^ T cells^15^. These two transcription factors are highly homologous (~60%); therefore, they compensate for each other in dendritic cells^28^. A recent study showed that BATF3 plays a role in Treg cells^18^; also, OX40L signaling increases IL-9 expression in Th9 cells^23^. In addition, OX40L signaling increases the expression of *Batf3*^24,25^. In light of this evidence, we investigated the role of BATF3 in Th9 cells. *Batf3* expression increased the production of IL-9 in Th9 cells; however, deletion of *Batf3* did not reduce the production of IL-9 under Th9 + OX40L conditions, which is a finding that is inconsistent with the overexpression results. This discrepancy can be explained by BATF compensating for BATF3 in *Batf3* KO cells; indeed, the transcription factors act redundantly in *Batf* and *Batf3* DKO cells. Our finding that knocking out *Batf* led to a marked reduction in the expression of IL-9 but that knocking out *Batf3* did not, and the finding that BATF induced the expression of *Il9* in DKO mice more strongly than BATF3 suggests that BATF has a stronger effect on IL-9 expression than BATF. Taken together, we propose that BATF3 and BATF play redundant roles during Th9 cell differentiation.

A compensatory role for BATF and BATF3 was confirmed in dendritic cells, and in Th2 and Th17 cells^15,34,35^. We conducted an overexpression study to determine whether BATF3 plays a compensatory role in Th9 cells. Overexpression of *Batf3* in *Batf* KO Th9 cells restored the expression of IL-9. To further study the compensatory role of Batf3, we designed in vivo experiments based on an adoptive transfer model. We found that *Batf* KO Th9 cells induced airway inflammation upon overexpression of BATF3. On the basis of these data, we propose that BATF and BATF3 play redundant roles, as BATF3 can compensate for the loss of BATF in Th9 cells.

BATF and IRF4 are transcription factors that are important for Th9 cell differentiation. In addition, BATF and IRF4 bind cooperatively to the *Il9* gene promoter^17^. Therefore, we asked whether the molecular mechanism was the same for BATF3. Co-IP experiments revealed that BATF3 interacts physically with IRF4, and a ChIP assay revealed that the BATF3–IRF4 complex binds to the *Il9* gene promoter. We also observed that BATF3 interacts with PU.1 (data not shown); however, the BATF3–PU.1 complex did not affect the activity of the *Il9* promoter. Therefore, the data suggest that BATF3 and BATF act via a similar mechanism to induce *Il9* expression.

BATF3 plays a role in other CD4 T cell subsets. In a previous study, we showed that BATF3 inhibits the differentiation of Treg cells^18^. Another study showed that OX40 signaling induced the expression of *Batf3* and inhibited the expression of *Foxp3*^25^. These studies suggest that one possible role of BATF3 in Th9 cells (in addition to the induction of IL-9 expression) is to inhibit the expression of *Foxp3* induced by TGF-β signaling. Thus, BATF3 may play multiple roles in Th9 differentiation.

In summary, we show that BATF3 induces the expression of IL-9 and that it plays a redundant role with BATF in Th9 cells. Taken together, the results provide valuable information that may be useful for developing treatments for allergies and asthma.

## Supplementary information

Supplementary information
