# Supplementary material for: BATF3 is sufficient for the induction of Il9 expression and can compensate for BATF during Th9 cell differentiation
Source: Exp Mol Med. 2019 Nov 27;51(11):143. doi: 10.1038/s12276-019-0348-6 (PMC6881360; doi:10.1038/s12276-019-0348-6)
Supplement: Supplementary file 1 — Supplementary information [file 12276_2019_348_MOESM1_ESM.docx]

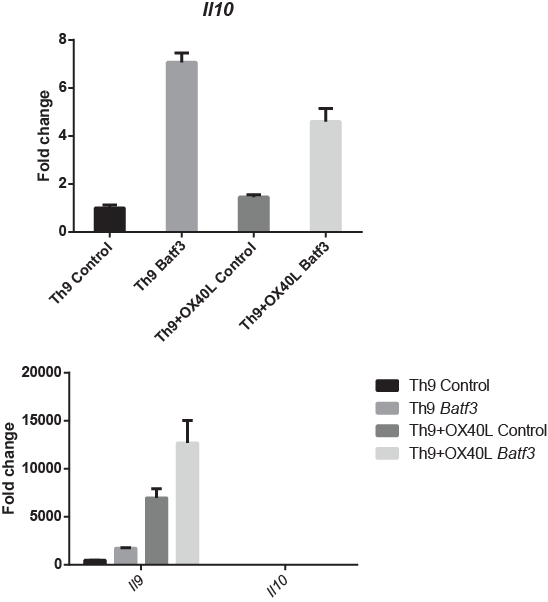


Figure S1. *Batf3* effect on *Il10* expression in Th9 cells

Expression of *Il10* was measured by qRT-PCR Batf3-overexpressing Th9 cells stimulated in presence or absence of OX40L.


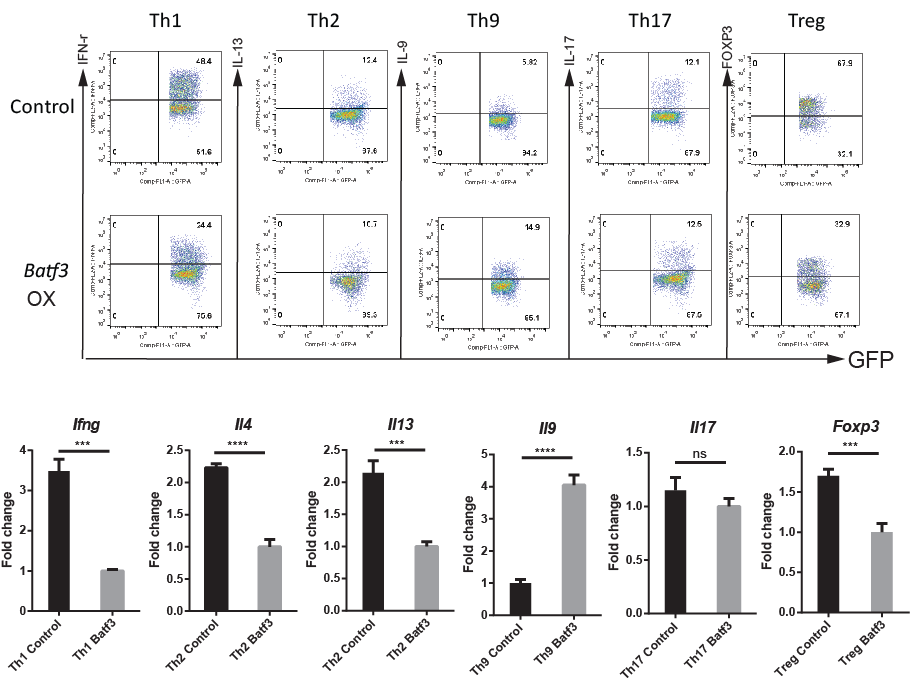


Figure S2. The effect of *Batf3* overexpression in each subset of CD4 T cells.

Naïve CD4 T cells were transduced with empty or *Batf3* expressing vector (OX) and stimulated under Th1, Th2, Th9, Th17, Treg differentiation conditions, and then analyzed by flow cytometry. Expression of mRNA was measured by qRT-PCR. P-values were calculated using Student’s t-test. *P<0.05, **P<0.01, and ***P<0.001. Experiments were repeated independently three times.


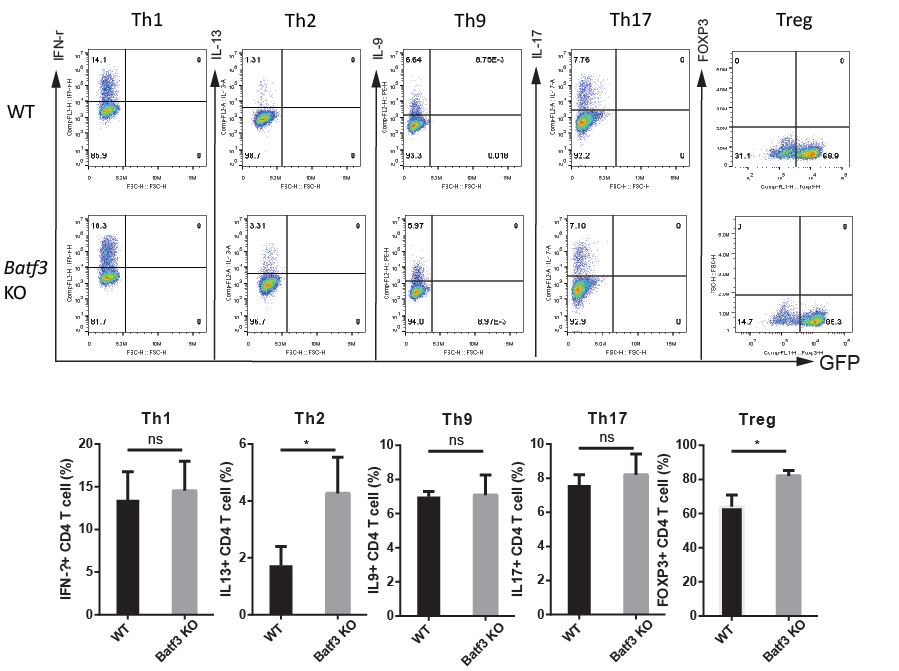


Figure S3. The effect of *Batf3* deletion in each subset of CD4 T cells.

Naïve CD4 T cells from WT and *Batf3* KO mice were stimulated under Th1, Th2, Th9, Th17, Treg differentiation conditions, and then analyzed by flow cytometry. Percentages of cytokine expressing cells are plotted at the bottom panel. P-values were calculated using Student’s t-test. *P<0.05. Experiments were repeated independently three times.

Table S1. Primer sequence of *Il9* CNS regions

| ChIP primers | Sequence | Locations |
| --- | --- | --- |
| *Il9* CNS0 | 5’-ATGCGGAATGGGTTTTCACT-3’  5’-AAGCTCCACACACTTAGTTTGT-3’ | -6287 to -6093 |
| *Il9* CNS1 | 5’-CCCTGTAACTCACTGTCTATCAGC-3’  5’-GCAGGAATTCTGGTTGTGAG-3’ | -375 to -270 |
| *Il9* CNS1a | 5′-CAGTCTACCAGCATCTTCCAGTCTAGC-3′  5′-GTGGGCACTGGGTATCAGTTTGATGTC-3′ | -20 |
| *Il9* CNS1b | 5′-GTCACTTGACAAAGGCTGTCTTATGCC-3′  5′-CAGAACCCGACTATTTGAAGAGCATC-3 | -159 |
| *Il9* CNS2 | 5’-TCACCCACTTTAGTCCTTTCAAAA-3’  5’-AATTACAGAATTTTGCCCCAGGTCCTG-3’ | +4888 to +4983 |
| *Il9* CNS2a | 5′-AATTACAGAATTTTGCCCCAGGTCCTG-3′  5′-GTTAATGCACAATTCATGTGCCAATCC-3′ | +4969 |
| *Il9* gene | 5’-TGATTGTACCACACCGTGCT-3’  5’-TATCCTTTTCACCCGATGGA-3’ | +1557 to +1657 |
